# *SimBoost*: a read-across approach for predicting drug–target binding affinities using gradient boosting machines

**DOI:** 10.1186/s13321-017-0209-z

**Published:** 2017-04-18

**Authors:** Tong He, Marten Heidemeyer, Fuqiang Ban, Artem Cherkasov, Martin Ester

**Affiliations:** 1 School of Computing Science, Simon Fraser University, 8888 University Drive, Burnaby, BC V5A 1S6 Canada; 2 Faculty of Medicine, Vancouver Prostate Center, University of British Columbia, Vancouver, BC V6H 3Z6 Canada

**Keywords:** Read-across, Gradient boosting, Drug–target interaction, Prediction interval, Applicability Domain, QSAR

## Abstract

Computational prediction of the interaction between drugs and targets is a standing challenge in the field of drug discovery. A number of rather accurate predictions were reported for various binary drug–target benchmark datasets. However, a notable drawback of a binary representation of interaction data is that missing endpoints for non-interacting drug–target pairs are not differentiated from inactive cases, and that predicted levels of activity depend on pre-defined binarization thresholds. In this paper, we present a method called *SimBoost* that predicts continuous (non-binary) values of binding affinities of compounds and proteins and thus incorporates the whole interaction spectrum from true negative to true positive interactions. Additionally, we propose a version of the method called *SimBoostQuant* which computes a prediction interval in order to assess the confidence of the predicted affinity, thus defining the Applicability Domain metrics explicitly. We evaluate *SimBoost* and *SimBoostQuant* on two established drug–target interaction benchmark datasets and one new dataset that we propose to use as a benchmark for read-across cheminformatics applications. We demonstrate that our methods outperform the previously reported models across the studied datasets.

## Background

Finding a compound that selectively binds to a particular protein is a highly challenging and typically expensive procedure in the drug development process, where more than 90% of candidate compounds fail due to cross-reactivity and/or toxicity issues. It is therefore an important topic in drug research to gain knowledge about the interaction of compounds and target proteins through computational methods. Such in silico approaches are capable of speeding up the experimental wet lab work by systematically prioritizing the most potent compounds and help predicting their potential side effects.

Recent studies [1] have demonstrated that machine learning-based approaches have the potential to predict compound-protein interactions on a large scale by learning from limited interaction data supplemented with information on the similarity among compounds and among proteins. Incorporating the similarity between drugs and between targets to infer the interaction of untested drug–target pairs is the essence of the read-across methodology [2].

The datasets commonly used for the training and evaluation of such machine learning-based prediction methods are the Enzymes, Ion Channels, Nuclear Receptor, and G Protein-Coupled Receptor datasets [3]. These datasets contain binary labels $Y_{(i,j)} = 1$ if drug–target pair $(d_{i} ,t_{j} )$ is known to interact (as shown by wet lab experiments) and $Y_{(i,j)} = 0$ if either $(d_{i} ,t_{j} )$ is known to not interact or if the interaction of $(d_{i} ,t_{j} )$ is unknown. The datasets tend to be biased towards drugs and targets that are considered to be more important or easier to test experimentally. As elaborated in [4], the use of such binary datasets has two major limitations: (1) true-negative interactions and missing values are not differentiated, and (2) a given compound-target interaction is treated as a binary on–off relationship, although it is more informative to use a continuous value that quantifies how strongly a compound binds to a target.

The study of [4] introduces two continuous interaction datasets and the continuous evaluation metric $CI$ and presents a read-across method *KronRLS* which predicts continuous binding affinities. The prediction in *KronRLS* is based on a similarity score for each drug–target pair, where the similarity of drug–target pairs is defined through the Kronecker product of a drug–drug similarity matrix and a target–target similarity matrix. Another method that has previously been shown to achieve high performance in drug target interaction prediction is Matrix Factorization (MF) [5–7], which in its simplest formulation learns to predict drug target interaction just from the given binding values without incorporating similarity information among drugs and among targets.

Intuitively both *KronRLS* and *MF* share the limitation of capturing only linear dependencies in the training data. To the best of our knowledge, no non-linear methods for drug–target interaction prediction have been presented in the literature. Furthermore, we believe that due to the biased nature of the training datasets it is necessary to assign a confidence score to a prediction. As emphasized in [8] it is important to address the uncertainty of the predictions of read-across approaches, but previous methods have neglected this need.

In this paper, we propose a novel non-linear method, *SimBoost*, for continuous drug–target binding affinity prediction, and a version *SimBoostQuant,* using quantile regression to estimate a prediction interval as a measure of confidence. Given a training dataset of continuous binding affinities and the similarities among drugs and among targets, *SimBoost* constructs features for drugs, targets, and drug–target pairs, and uses gradient boosting machines to predict the binding affinity for a drug–target pair and to generate a prediction interval. Besides gradient boosting, another non-linear method that can predict the value of some dependent variable and generate a prediction interval is random forests [9]. We have two reasons for choosing gradient boosting over random forests. First, all the trees in a random forest can be seen as identically distributed. Thus, if their prediction is biased then the average of the prediction is also biased, which may lead to a less accurate final result. Second, the random forest algorithm for quantile regression introduced in [9] produces the same tree structure as the usual random forest algorithm and only changes the way in which predictions are generated for the leaf nodes. This implies that the trees are not grown in a shape optimized for quantile regression. Our proposed gradient boosting method overcomes both limitations.

Gradient boosting machines have been employed in previous QSAR studies [10, 11]. Svetnik et al. [11] compares the performance of gradient boosting machines against commonly used QSAR methods such as support vector machines for regression and classification problems involving only compounds. Singh and Shikha [10] utilizes gradient boosting machines to predict toxic effects of nanomaterials. In both studies, gradient boosting machines show promising results in terms of prediction performance, speed and robustness. A major difference of our work compared to these previous studies is the problem formulation: In [10, 11] a prediction is made for a single entity (nanomaterial or compound), and descriptors for the compounds/nanomaterials are given. In the drug–target setting, on the other hand, we make predictions for pairs of entities, i.e. one drug and one target. Therefore, we present a novel feature engineering step on which our method relies in the learning and prediction phases.

## Related work

Traditional methods for drug target interaction prediction typically focus on one particular target of interest. These approaches can again be divided into two types which are target-based approaches [12–14] and ligand-based approaches [15–18]. In target-based approaches the molecular docking of a candidate compound with the protein target is simulated, based on the 3D structure of the target (and the compound). This approach is widely utilized to virtually screen compounds against target proteins; however this approach is not applicable when the 3D structure of a target protein is not available which is often the case, especially for G-protein coupled receptors and ion channels. The intuition in ligand-based methods is to model the common characteristics of a target, based on its known interacting ligands (compounds). One interesting example for this approach is the study [4] which utilizes similarities in the side-effects of known drugs to predict new drug–target interactions. However, the ligand-based approach may not work well if the number of known interacting ligands of a protein target is small.

To allow more efficient predictions on a larger scale, i.e. for many targets simultaneously, and to overcome the limitations of the traditional methods, machine learning based approaches have attracted much attention recently. In the chemical and biologicals sciences, machine learning-based approaches have been known as (multi-target) Quantitative structure–activity relationship (QSAR) methods, which relate a set of predictor variables, describing the physico-chemical properties of a drug–target pair, to the response variable, representing the existence or the strength of an interaction.

Current machine learning methods can be classified into two types, which are feature-based and similarity-based approaches. In feature-based methods, known drug–target interactions are represented by feature vectors generated by combining chemical descriptors of drugs with descriptors for targets [19–23]. With these feature vectors as input, standard machine learning methods such as Support Vector Machines (SVM), Naïve Bayes (NB) or Neural Networks (NN) can be used to predict the interaction of new drug–target pairs. Vina et al. [24] proposes a method taking into consideration only the sequence of the target and the chemical connectivity of the drug, but without relying on geometry optimization or drug–drug and target–target similarities. Cheng et al. [25] introduces a multi-target QSAR method that integrates chemical substructures and protein sequence descriptors to predict interactions for G-protein coupled receptors and kinases based on two comprehensive data sets derived from the ChEMBL database. Merget et al. [26] evaluates different machine learning methods and data balancing schemes and reports that random forests yielded the best activity prediction and allowed accurate inference of compound selectivity.

In similarity-based methods [3, 27–32], similarity matrices for both the drug–drug pairs and the target–target pairs are generated. Different types of similarity metrics can be used to generate these matrices [33]; typically, chemical structure fingerprints are used to compute the similarity among drugs and a protein sequence alignment score is used for targets. One of the simplest ways of using the similarities is a Nearest Neighbor classifier [28], which predicts new interactions from the weighted (by the similarity) sum of the interaction profiles of the most similar drugs/targets. The Kernel method proposed in [27] computes a similarity for all drug–target pairs (a pairwise-kernel) using the drug–drug and target–target similarities and then uses this kernel of drug–target pairs with known labels to train an SVM-classifier. The approaches presented in [28–30] represent drug–target interactions by a bipartite graph and label drug–target pairs as +1 if the edge exists or −1, otherwise. For each drug and for each target, a separate SVM (local model) is trained, which predicts interactions of that drug (target) with all targets (drugs). The similarity matrices are used as kernels for those SVMs, and the final prediction for a pair is obtained by averaging the scores for the respective drug SVM and target SVM.

All of the above machine-learning based methods for drug–target interaction prediction formulate the task as a binary classification problem, with the goal to classify a given drug–target pair as binding or non-binding. As pointed out in [4], drawbacks of the binary problem formulation are that true-negative interactions and untested drug–target pairs are not differentiated, and that the whole interaction spectrum, including both true-positive and true-negative interactions, is not covered well. Pahikkala et al. [4] introduces the method KronRLS which predicts continuous drug–target binding affinity values. To the best of our knowledge, KronRLS is the only method in the literature which predicts continuous binding affinities, and we give a detailed introduction to KronRLS below, since we use it as baseline in our experiments. Below, we also introduce Matrix Factorization as it was used in the literature for binary drug–target interaction prediction and as it plays an important role in our proposed method.

### *KronRLS*

Regularized Least Squares Models (RLS) have previously been shown to be able to predict binary drug target interaction with high accuracy [31]. *KronRLS* as introduced in [4] can be seen as a generalization of these models for the prediction of continuous binding values. Given a set $\{ d_{i} \}$ of drugs and a set $\{ t_{j} \}$ of targets, the training data consists of a set $X = \{ x_{1} , \ldots ,x_{m} \}$ of drug–target pairs ($X$ is a subset of $\{ d_{i} \times t_{j } \}$) and an associated vector $y = y_{1} , \ldots ,y_{m}$ of continuous binding affinities. The goal is to learn a prediction function $f(x)$ for all possible drug–target pairs $x \in \{ d_{i} \times t_{j } \}$, i.e. a function that minimizes the objective:

$$J(f) = \mathop \sum \limits_{i = 1}^{m} (y_{i} - f(x_{i} ))^{2} + \lambda ||f||_{k}^{2}$$

In the objective function, $||f||_{k}^{2}$ is the norm of $f$, which is associated to a kernel function $k$ (described below), and $\lambda > 0$ is a user defined regularization parameter. A minimizer of the above objective can be expressed as

$$f(x) = \mathop \sum \limits_{i = 1}^{m} a_{i} k(x,x_{i} )$$

The kernel function $k$ is a symmetric similarity measure between two of the $m$ drug–target pairs, which can be represented by an $m \times m$ matrix $K$. For two individual similarity matrices $K_{d}$ and $K_{t}$ for the drugs and targets respectively, a similarity matrix for each drug–target pair can be computed as $K_{d} \otimes K_{t}$, where $\otimes$ stands for the Kronecker product. If the training set $X$ contains every possible pair of drugs and targets, $K$ can be computed as $K = K_{d} \otimes K_{t}$ and the parameter vector $a$ can be learnt by solving the following system of linear equations:

$$(K + \lambda I)a = y$$

where $I$ is the $d_{i} \times t_{j}$ identity matrix. If only a subset of $\{ d_{i} \times t_{j} \}$ is given as training data, the vector $y$ has missing values. To learn the parameter $a$, [4] suggests to use conjugate gradient with Kronecker algebraic optimization to solve the system of linear equations.

### Matrix factorization

The Matrix Factorization (MF) technique has been demonstrated to be effective especially for personalized recommendation tasks [34], and it has been previously applied for drug–target interaction prediction [5–7]. In MF, a matrix $M \in R^{d \times t}$ (for the drug–target prediction task, $M$ represents a matrix of binding affinities of $d$ drugs and $t$ targets) is approximated by the product of two latent factor matrices $P \in R^{k \times d}$ and $Q \in R^{k \times t}$.

The factor matrices $P$ and $Q$ are learned by minimizing the regularized squared error on the set of observed affinities $\kappa$:

$$\mathop {min}\limits_{Q,P} \mathop \sum \limits_{{(d_{i} ,t_{j} ) \in \kappa }}^{{}} (m_{i,j} - q_{i}^{T} p_{j} )^{2} + \lambda (||p||^{2} + ||q||^{2} )$$

The term $(m_{i,j} - q_{i}^{T} p_{j} )^{2}$ represents the fit of the learned parameters to the observed binding affinities. The term $\lambda (||p||^{2} + ||q||^{2} )$ penalizes the magnitudes of the learned parameters to prevent overfitting, and the constant $\uplambda$ controls the weight of the two terms. With learned matrices $P$ and $Q$, a matrix $M^{{\prime }}$ with predictions for all drug–target pairs can be computed as:

$$M^{{\prime }} = P^{T} Q$$

In *SimBoost*, the columns of the factor matrices $P$ and $Q$ are utilized as parts of the feature vectors for the drugs and targets respectively and thus Matrix Factorization is used as a feature extraction step.

## Methods

### Problem definition

We assume input data in the format $(M,D,T)$, where $M$ is a matrix with continuous values where $M_{i,j}$ represents the binding affinity of drug $i$ and target $j$. $D$ is a similarity matrix of drugs, and $T$ is a similarity matrix of targets. Specifically, we define $M_{i, \cdot }$ as the $i$-th row of $M$, and $M_{ \cdot ,j}$ as the $j$-th column of $M$. Similarly, we define $D_{i, \cdot }$ as the $i$-th row of $D$, and $T_{ \cdot ,j}$ as the $j$-th row of $T$. Only a subset of the elements of $M$ is observed, and our goal is to predict all the non-observed values in $M$ with the given information.

### *SimBoost* and *SimBoostQuant*

Our proposed method, called *SimBoost*, constructs features for each drug, each target and each drug–target pair. These features represent the properties of drugs, targets and drug–target pairs, respectively. *SimBoost* associates a feature vector with each pair of one drug and one target. From pairs with observed binding affinities, it trains a gradient boosting machine model to learn the nonlinear relationships between the features and the binding affinities. Once the model is trained, *SimBoost* can make predictions of the binding affinities for unobserved drug–target pairs, based on their known features.

We also propose a version of *SimBoost*, called *SimBoostQuant*, which computes the confidence of the prediction by using quantile regression to learn a prediction interval for a given drug–target pair as a measure of the confidence of the prediction.

**Fig. 1:**
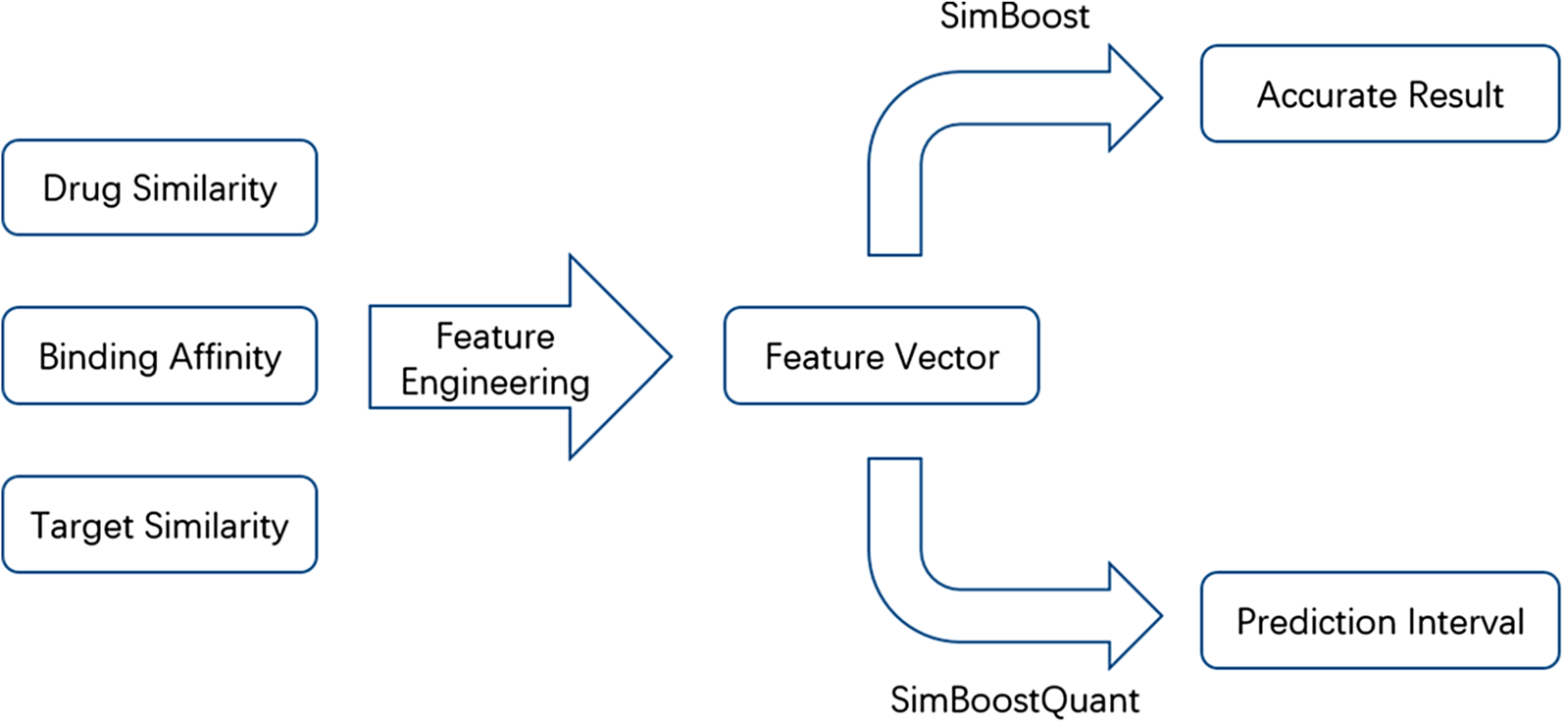
The workflow of *SimBoost* and *SimBoostQuant*

Figure 1 illustrates the workflow of *SimBoost* and *SimBoostQuant*, consisting of the three steps of feature engineering, gradient boosting trees and prediction interval. These steps are introduced in the following.

### Feature engineering

We define three types of features to describe the properties of drugs, targets and drug–target pairs.

Type 1 features: We extract features for each single object (i.e. drug or target).

- Number of observations in $M$ for the object (n.obs).
  - The number of observations in the corresponding row/column of $M$.
- Average of all similarity scores of the object (ave.sim).
  - For drug $i$, the average of $D_{i, \cdot }$.
  - For target $j$, the average of $T_{j, \cdot }$.
- Histogram of the similarity values of the object (hist.sim).
  - A vector of frequencies of the similarity values, where the number of bins is an input parameter.
- Average of the observed values for the object in $M$ (ave.val).
  - For drug $i$, the average of $M_{i, \cdot }$.
  - For target $j$, the average of $M_{ \cdot ,j}$.

Type 2 features: We build two networks, one for drugs and another one for targets, from $D$ and $T$, respectively. The nodes are drugs or targets, and an edge between two nodes exists if their similarity is above a user-defined threshold. We define the following features for each node.

- Number of neighbours (num.nb).
- The similarity values of the $k$-nearest neighbours of the node (k.sim).
- The average of the Type 1 features among the $k$-nearest neighbours of the node (k.ave.feat), simply averaging these vectors from different objects, which results in a vector of the same length.
- The average of the Type 1 features among the $k$-nearest neighbours of the node, weighted by the similarity values (k.w.ave.feat).
- Betweenness, closeness and eigenvector centrality of the node as introduced in [35] (bt, cl, ev).
- PageRank score as described in [36] (pr).

Type 3 features: We build a network for drugs and targets from $M$. The nodes are drugs and targets, and an edge connects a drug and a target, with the binding affinity as the edge weight. We define the following features for each drug–target pair.

- Latent vectors from matrix factorization (mf).
  - The latent vector for the drug or the target, obtained by matrix factorization of $M$.
- Weighted scores from drug to target’s neighbours (if any) (d.t.ave).
  - If drug $i$ has observed affinities with target $j$‘s neighbours, average the values.
- Weighted scores from target to drug’s neighbours (if any) (t.d.ave).
  - If target $j$ has observed affinities with drug $i$‘s neighbours, average the values.
- Betweenness, closeness and eigenvector centrality of the node (d.t.bt, d.t.cl, d.t.ev).
- PageRank score (d.t.pr).

For each drug and target we build Type 1 and Type 2 feature vectors, and for each drug–target pair we build a Type 3 feature vector. For each drug–target pair $(d_{i} ,t_{j} )$, we build a feature vector by concatenating the Type 1 and Type 2 feature vectors for $d_{i}$ and $t_{j}$, and the Type 3 feature vector for $(d_{i} ,t_{j} )$, as illustrated in Table 1.

**Table 1:** Structure of feature vector for $(d_{i} ,t_{j} )$

Type 1 of *d*_*i*_	Type 1 of *t*_*j*_	Type 2 of *d*_*i*_	Type 2 of *t*_*j*_	Type 3 of $(d_{i} ,t_{j} )$

### Gradient boosting regression trees

To predict the continuous binding affinity for drug–target pairs, we train a supervised learning model based on the features defined in the Feature Engineering section. We choose the gradient boosting machine as our model, which was originally proposed in [37], because of its following benefits [38, 39]:

- Accuracy: the boosting algorithm is an ensemble model, which trains a sequence of “weak learners” to gradually achieve a good accuracy.
- Efficiency: the training process can be parallelized, greatly reducing the training time.

In the following, we provide a brief introduction to a variant of this model, gradient boosting regression trees, which we use in our methods. The details are described in [38, 39]. In the common supervised learning scenario, the data set can be represented by a set containing $n$ paired feature vectors and labels: $D = \{ (x_{i} ,y_{i} )\}$ ($|D| = n$). In the context of our task, $x_{i} \in R^{d}$ is the vector of features of the $i$-th drug–target pair, while $y_{i} \in R$ is its binding affinity.

In the gradient boosting regression trees model, the score $\hat{y}_{i}$ predicted for input $x_{i}$ is given by the following functional form:

$$\hat{y}_{i} = \phi (x_{i} ) = \mathop \sum \limits_{k = 1}^{K} f_{k} (x_{i} ), f_{k} \in F$$

where $K$ is the number of regression trees and $F$ is the space of all possible trees.

To learn the set of trees $\{ f_{k} \}$, we define the following regularized objective function:

$$L(\phi ) = \sum\limits_{i} {l(\hat{y}_{i} , y_{i} )} + \sum\limits_{k} {\varOmega (f_{k} )}$$

where $l$ is a differentiable loss function that evaluates the prediction $\hat{y}_{i}$ with regard to the known binding affinity $y_{i}$. The second term $\varOmega$ measures the complexity of the model (i.e., the set of trees) to avoid overfitting. With this objective function, a simple and predictive set of boosted trees will be selected as the best model.

Because the model includes trees as parameters, we cannot directly use traditional optimization methods in Euclidean space to find the solution. Instead, we train the model additively: at each iteration $t$, $F$ is searched to find a new tree $f_{t}$ that optimizes the objective function, which is then added to the ensemble. Formally, let $\hat{y}_{i}^{(t)}$ be the prediction for the $i$-th pair at the $t$-th iteration, the model finds $f_{t}$ that optimizes the following objective.

$$L^{(t)} = \mathop \sum \limits_{i = 1}^{n} l(y_{i} , \hat{y}_{i}^{(t)} ) + \mathop \sum \limits_{i = 1}^{t} \varOmega (f_{i} ) = \mathop \sum \limits_{i = 1}^{n} l(y_{i} ,\hat{y}_{i}^{(t - 1)} + f_{t} (x_{i} )) + \mathop \sum \limits_{i = 1}^{t} \varOmega (f_{i} )$$

This objective means that the model adds the best function to the set. In the general setting, the above objective is still hard to optimize, and we approximate the objective using the second order Taylor expansion.

$$L^{(t)} \simeq \mathop \sum \limits_{i = 1}^{n} \left[l(y_{i} ,\hat{y}_{i}^{(t - 1)} ) + g_{i} f_{t} (x_{i} ) + \frac{1}{2}h_{i} f_{t}^{2} (x_{i} )\right] + \mathop \sum \limits_{i = 1}^{t} \varOmega (f_{i} )$$

where $g_{i} = \partial_{{\hat{y}^{(t - 1)} }} l(y_{i} ,\hat{y}_{i}^{(t - 1)} )$ and $h_{i} = \partial_{{\hat{y}^{(t - 1)} }}^{2} l(y_{i} ,\hat{y}_{i}^{(t - 1)} )$. We can remove the constant terms to obtain the following approximate objective at step $t$:

$$\tilde{L}^{(t)} = \mathop \sum \limits_{i = 1}^{n} \left[ {g_{i} f_{t} (x_{i} ) + \frac{1}{2}h_{i} f_{t}^{2} (x_{i} )} \right] + \varOmega (f_{t} )$$

A gradient boosting algorithm iteratively adds trees that optimize $\tilde{L}^{(t)}$ for a number of user-specified iterations.

Usually in supervised learning tasks for continuous data, we use the squared loss function $L = (y - \hat{y})^{2}$ to compute the error. The first and second order gradient of this loss function are:

$$g_{i} = 2(\hat{y} - y)$$

$$h_{i} = 2$$

We define $\varOmega$ as follows:

$$\varOmega \left( {f_{t} } \right) = \gamma T + \frac{1}{2}\lambda \mathop \sum \limits_{j}^{T} w_{j}^{2}$$

where ${\text{T}}$ is the number of trees, $w_{j}^{2}$ is the prediction score for data corresponding to the $j$-th leaf from $f_{t}$ and $\gamma$ and $\lambda$ are two weight parameters.

### Prediction intervals

We extend gradient boosting regression trees by the concept of quantile regression to characterize the confidence of the prediction. Suppose the model can predict the quantile given the quantile parameter $\upalpha$. To obtain the interval, we need to train the model twice to calculate the $\upalpha$ quantile and the ($1 -\upalpha$) quantile to get the boundary of the prediction interval. To make a prediction for binding affinity, we use the median of the interval.

To perform quantile regression, we use the following loss function instead of the squared loss:

$$l_{i} = \alpha (y_{i} - u_{i} )I_{{y_{i} > u_{i} }} + (1 - \alpha )(y_{i} - u_{i} )I_{{y_{i} < u_{i} }}$$

where $\alpha$ is the quantile parameter, $y_{i}$ is the true binding affinity, and $u_{i}$ is the prediction.

Within the framework of gradient boosting trees, the new loss function can be optimized with stochastic gradient descent. The gradient for each data sample is

$$g_{i} = \alpha I_{{y_{i} > u_{i} }} - (1 - \alpha )I_{{y_{i} < u_{i} }}$$

The second order gradient is not applicable here, therefore we will set it to 1.

Since we care more about drug–target pairs with high binding affinities, the model weights these drug–target pairs more. To achieve that, we weight the prediction error by the true affinity, i.e. the larger the true affinity is the more weight the prediction error gets. The resulting loss function and the gradient are as follows:

$$l_{i} = y_{i} [\alpha (y_{i} - u_{i} )I_{{y_{i} > u_{i} }} + (1 - \alpha )(y_{i} - u_{i} )I_{{y_{i} < u_{i} }} ]$$

$$g_{i} = y_{i} [\alpha I_{{y_{i} > u_{i} }} - (1 - \alpha )I_{{y_{i} < u_{i} }} ]$$

## Experiments

### Data

We evaluated the performance of the proposed methods on three drug–target binding affinity datasets. We used the two large-scale biochemical selectivity assays for clinically relevant kinase inhibitors from the studies by [40, 41] that were already used to evaluate the performance of *KronRLS* in the original paper [4]. These two datasets will be referred to as the *Davis* and the *Metz* dataset. The *Davis* dataset is fully populated with binding affinities observed for all pairs of 68 drugs and 442 targets, measured by the $K_{d }$ value (kinase dissociation constant). The *Metz* dataset consists of 1421 drugs and 156 targets, and for 42% of the drug–target pairs the binding affinity is given as the $pK_{i}$ value (log transformed kinase inhibition constant). $K_{d }$ values in the *Davis* dataset were transformed into logspace ($pK_{d}$) as:

$$pK_{d} = - log_{10} \left( {\frac{{K_{d} }}{1e9}} \right)$$

The *Davis* and *Metz* datasets are suitable for the evaluation of predictive models for drug–target interaction because data heterogeneity is not an issue. We can assume that the experimental settings for the measured drug–target pairs in each dataset were the same and the binding affinities are comparable. When working with experimental results that come from multiple sources the data might be heterogeneous: In one case the binding affinity might be measured by $K_{i}$, in another case by $K_{d}$ and in a third case by $IC_{50}$. Another source of data heterogeneity are different experimental settings. An approach to integrate observations from different sources, named *KIBA*, and a corresponding dataset are presented in [42]. In this work the authors integrated the experimental results from multiple databases into a bioactivity matrix of 52,498 compounds and 467 targets, including 246,088 observations. The binding affinities in this matrix are given as *KIBA*-values. We used this dataset to obtain a third evaluation dataset, which we call the *KIBA* dataset, by removing all drugs and targets with less than 10 observations from the original dataset (downloaded from the supplementary materials of [42]), resulting in a dataset of 2116 drugs and 229 targets with a density of 24%.

**Fig. 2:**
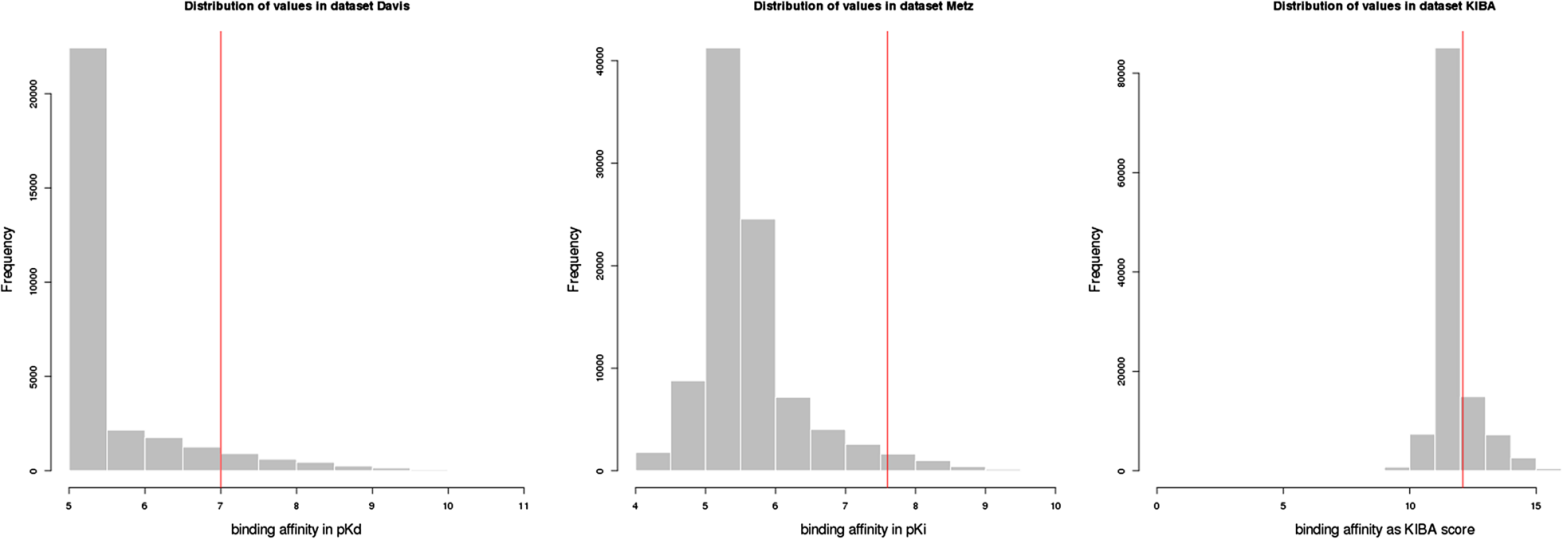
Distribution of values in the three datasets (*Davis*, *Metz* and *KIBA* from *left* to *right*) and binarization thresholds (*vertical red line*)

For the evaluation of the model we also include a performance evaluation for the classification of drug–target pairs into binding or non-binding, using the metrics AUC and AUPR. For these experiments, we used versions of the datasets binarized by applying thresholds as done in [4]. For the *Metz* dataset we used the threshold of $pK_{i} \ge 7.6$ as suggested in [4] to assign a label of $1$, i.e. binding. For the *Davis* dataset we used a threshold of $pK_{d} \ge 7.0$ which is a bit less stringent than the threshold suggested in [4]. In the *KIBA* dataset, the lower the *KIBA*-score, the higher the binding affinity, and [42] suggests a threshold of *KIBA value* ≤3.0 to binarize the dataset. In an additional preprocessing step, we transformed the *KIBA* dataset by taking the negative of each value and adding the minimum to all values in order to obtain a threshold where all values above the threshold are classified as binding. The *KIBA* threshold of 3.0 in the untransformed dataset then becomes 12.1. Table 2 lists the sizes and densities of the datasets, and Fig. 2 illustrates the distributions of the affinity values for the three datasets.

**Table 2:** The statistics of the three datasets

Dataset	Number of drugs	Number of targets	Density (%)
*Davis*	68	442	100
*Metz*	1421	156	42.1
*KIBA*	2116	229	24.4

In the original *Davis* dataset a large fraction of the values is given as “>10,000*K*_*d*_”, meaning no binding affinity was detected in the wet lab experiment. These values were transformed to 10,000*K*_*d*_ (5*pK*_*d*_) in the preprocessed dataset, which explains the high bar at value 5*pK*_*d*_.

As drug–drug and target–target similarity matrices for the *Davis* and *Metz* dataset we used the precomputed matrices that are provided on the website of [4]. Here, the drug–drug similarity was computed based on the 2D chemical structure of the compounds, using the structure clustering server at PubChem. This tool clusters the compounds based on the structure similarity using the single linkage algorithm [44] and allows to download a similarity matrix containing the similarity for each drug–drug pair. The target–target similarity was computed based on the protein sequences, using the normalized Smith-Waterman score [3]. For the *KIBA* dataset we obtained the drug–drug similarity matrix through the compound clustering tool of PubChem as done by the authors of [4] for the Davis and Metz datasets. The given ChEMBL IDs of the compounds were first matched to their PubChem CIDs which were then used as input to the PubChem web interface.

The web tool allows to download a similarity matrix for the compounds as described above (similarly as for the drug–drug similarity of the *Metz* and David datasets). For the *KIBA* dataset we downloaded the protein sequences from *NCBI* and computed the normalized Smith Waterman similarity for each pair by aligning the sequences using the *Biostrings* R package.

### Results

The baselines evaluated in our experiments are matrix factorization trained on continuous data (referred to as MF), and the *KronRLS* method trained on both continuous (referred to as Continuous *KronRLS*) or binarized data (referred to as Binary *KronRLS*). For MF, we use the implementation *libmf* as described in [43]. For *KronRLS*, we use the original code from the author of [4].

We also compare the performance of the two models we proposed. The difference lies in the loss function, the first one employs the usual squared loss (*SimBoost*), the second one employs the quantile regression loss (*SimBoostQuant*). We use the library *xgboost* to train the model [38].

We perform fivefold cross validation. To ensure that no target is used only for training or only for testing, we build the folds in a way such that every target has an observation in at least two folds. To test the variance of the performance scores, we repeat the cross validation ten times for each model on each dataset, and report the mean and standard deviation for each metric.

The evaluation metrics used are Root Mean Squared Error (RMSE), Area Under the Curve (AUC), Area Under the Precision-Recall curve (AUPR) and Concordance Index (CI). The RMSE is a commonly used metric for the error in continuous prediction. The binary metrics AUC and AUPR are commonly used in the related work on drug–target interaction prediction, measuring the ranking error of the predictions. The AUPR is commonly used in these types of studies because it punishes more false positive predictions in highly unbalanced data sets [45]. The CI is a ranking metric for continuous values that was suggested by [4]. The CI over a set of paired data is the probability that the predictions for two randomly drawn drug–target pairs with different label values are predicted in the correct order.

*KronRLS* determines the optimal regularization parameter by an inner cross validation step, and the parameter which gives the best performance on the desired metric is selected to predict the test fold. The prediction of *KronRLS* might therefore depend on the used metric. Specifically, when the classification metrics AUC or AUPR are applied, *KronRLS* learns and predicts binary labels, meaning that the datasets are binarized according to the cutoff threshold before the training step.

Our methods in contrast only predict continuous values, and the binarization threshold is applied after the prediction step to calculate the $AUC$ and $AUPR$ metrics. We argue that, given two models $A$ and $B$, where $A$ learns to predict continuous values and model $B$ learns to predict binary values, and the performance of model $A$ in terms of $AUC$ and $AUPR$ is as good as the performance of model $B$, model $A$ is advantageous because it does not need to be retrained when the threshold for the dataset is changed. For a fair comparison we also list the performance of *KronRLS* in terms of $AUC$ and $AUPR$ when continuous values were predicted and the threshold was applied after the prediction step.

## Discussion

The results with respect to the four performance metrics are presented in Table 3 (*Davis* dataset), Table 4 (*Metz* dataset) and Table 5 (*KIBA* dataset). For every metric, the value for the best-performing method is highlighted in bold font.

**Table 3:** Results on the *Davis* data set, with the mean and standard deviation from 10 repetitions

	RMSE	AUC	AUPR	CI
MF	0.509 ± 0.010	0.876 ± 0.004	0.499 ± 0.017	0.816 ± 0.004
Continuous *KronRLS*	0.608 ± 0.002	0.942 ± 0.001	0.679 ± 0.003	0.860 ± 0.001
Binary *KronRLS*	–	0.931 ± 0.001	0.686 ± 0.006	–
*SimBoost*	**0.247** ± 0.003	**0.956** ± 0.001	**0.758** ± 0.005	**0.884** ± 0.001
*SimBoostQuant*	0.36 ± 0.001	0.942 ± 0.002	0.680 ± 0.002	0.871 ± 0.004

**Table 4:** Results on the *Metz* data set, with the mean and standard deviation from 10 repetitions

	RMSE	AUC	AUPR	CI
MF	0.303 ± 0.005	0.895 ± 0.003	0.358 ± 0.011	0.788 ± 0.001
Continuous *KronRLS*	0.562 ± 0.001	0.943 ± 0.001	0.518 ± 0.003	0.789 ± 0.001
Binary *KronRLS*	–	0.932 ± 0.001	0.565 ± 0.004	–
*SimBoost*	**0.166** ± 0.001	**0.958** ± 0.001	**0.629** ± 0.003	**0.851** ± 0.001
*SimBoostQuant*	0.249 ± 0.002	0.942 ± 0.002	0.523 ± 0.004	0.813 ± 0.020

**Table 5:** Results on the *KIBA* data set, with the mean and standard deviation from 10 repetitions

	RMSE	AUC	AUPR	CI
MF	0.382 ± 0.003	0.831 ± 0.002	0.631 ± 0.004	0.792 ± 0.001
Continuous *KronRLS*	0.620 ± 0.001	0.884 ± 0.001	0.735 ± 0.001	0.792 ± 0.001
Binary *KronRLS*	–	0.904 ± 0.001	0.7660 ± 0.001	–
*SimBoost*	**0.204** ± 0.001	**0.907** ± 0.001	**0.782** ± 0.001	**0.847** ± 0.001
*SimBoostQuant*	0.299 ± 0.001	0.875 ± 0.001	0.708 ± 0.002	0.796 ± 0.001

We observe that *SimBoost* consistently outperforms all baselines on all datasets in terms of all performance metrics. Based on the standard deviation obtained from 10 repetitions, the improvement is significant (Table 4).

In particular, for the *Davis* dataset, *SimBoost* reduces the RMSE of MF by 51% and improves the AUPR of Continuous *KronRLS* by 12%. For the *Metz* dataset, *SimBoost* reduces the RMSE of MF by 45%, improves the AUPR of Binary *KronRLS* by 11% and improves the CI of MF by 8%. For the *KIBA* dataset, *SimBoost* reduces the RMSE of the MF by 47%, improves the AUPR of Binary *KronRLS* by 2% and improves the CI of MF by 7%.

*SimBoostQuant* achieves the second best performance in terms of RMSE, AUC and CI. While this model is not as good as *SimBoost* in terms of prediction performance, it has the advantage of quantifying the confidence of the predicted value.

**Fig. 3:**
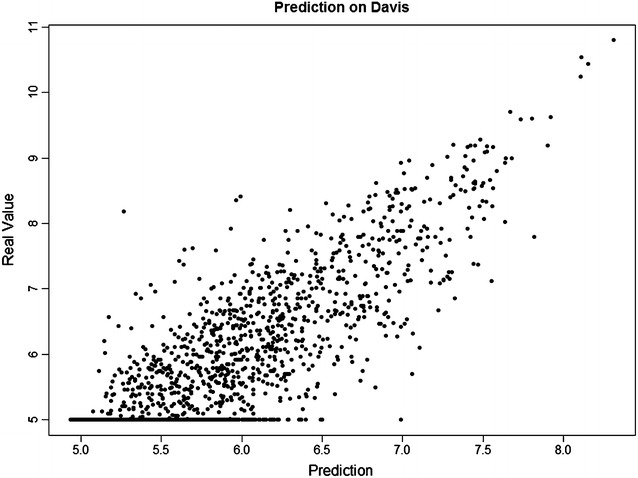
Prediction from *SimBoost* against the real values on *Davis*

**Fig. 4:**
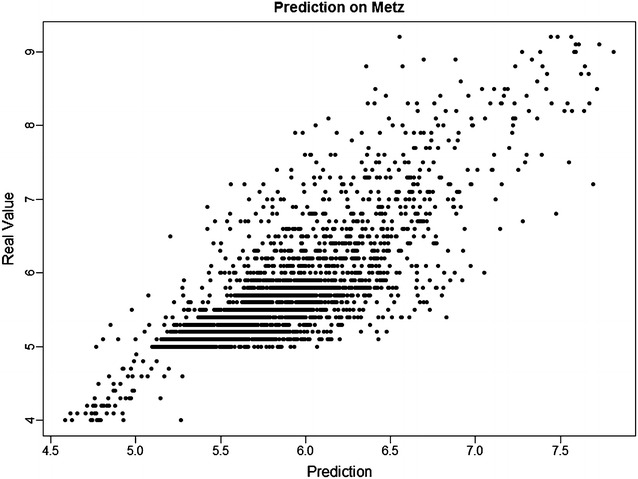
Prediction from *SimBoost* against the real values on *Metz*

**Fig. 5:**
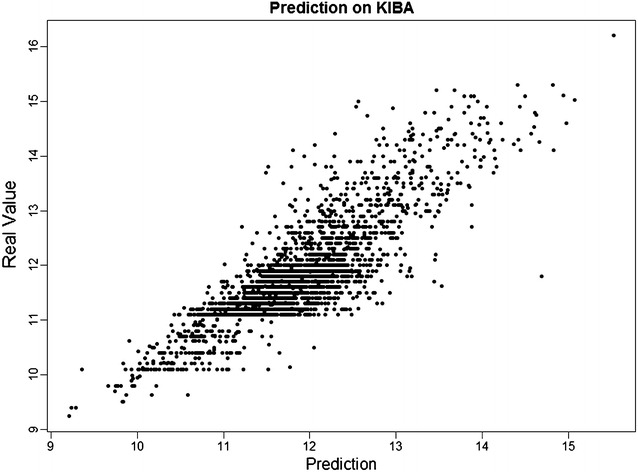
Prediction from *SimBoost* against the real values on *KIBA*

To provide further insight into the performance of *SimBoost*, Figs. 3, 4 and 5 plot the predictions of *SimBoost* against the actual values on the three datasets.

**Fig. 6:**
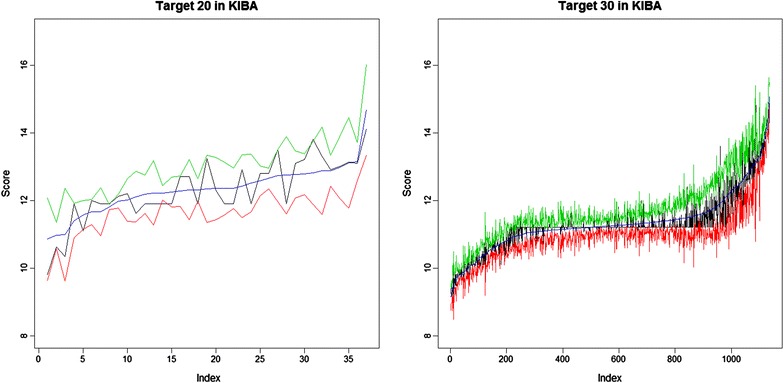
The prediction intervals of two targets from *KIBA*

Figure 6 illustrates the prediction intervals from *SimBoostQuant* for all drugs for two targets from the *KIBA* dataset.

The highest dash-dot green line corresponds to the upper quantile, the lowest dashed red line corresponds to the lower quantile. The dotted blue line in the middle is the average of the quantiles, used as our prediction. The solid black line is the true value in the training dataset. The drugs are listed on the x-axis, sorted by the predicted value.

**Fig. 7:**
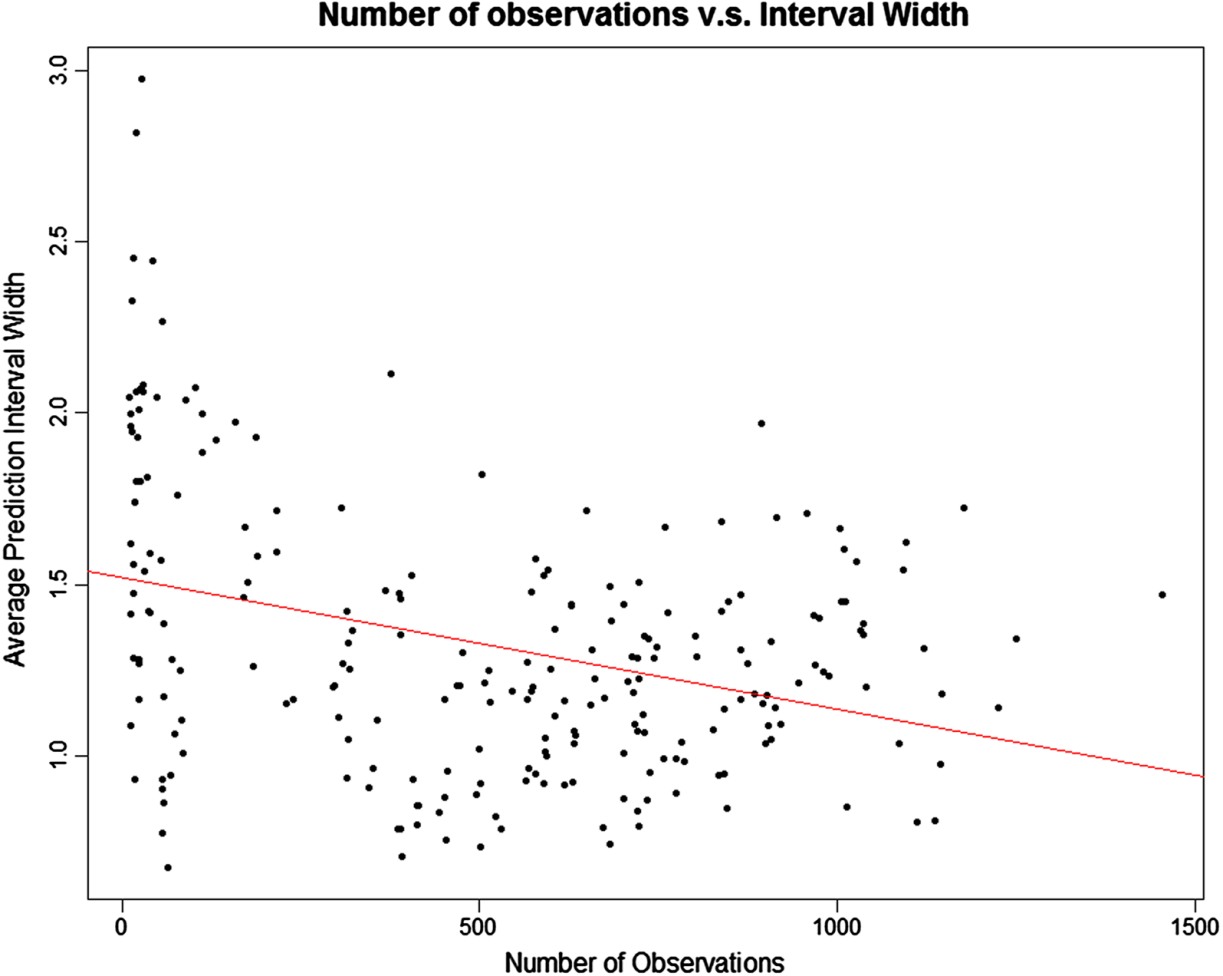
The relationship between the number of observations and the average width of the prediction intervals, in the *KIBA* dataset

The numbers of observations for drugs in the two plots from left to right are 37 and 1136, and the average interval width is 1.51 and 0.75. We observe that the width decreases as the number of observations increases, which is demonstrated in Fig. 7.

This is because generally the more observations, the more information has the model. For a given target, the width of the prediction interval varies a lot for different compounds, therefore among the compounds with high predicted affinities we choose those with narrower intervals.

**Fig. 8:**
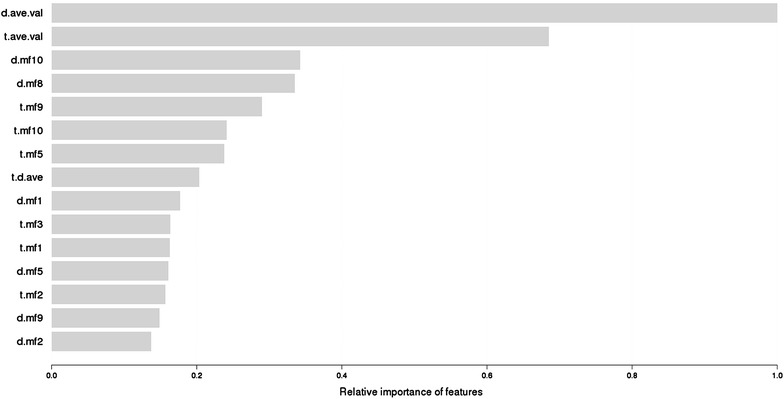
Relative feature importance in *Davis*

Figure 8 plots the relative importance of the features, as computed by the xgboost package with feature names as introduced in the “Methods” section.

We note that the most important features are the average affinity of the drug and the target, as well as the latent factors obtained from matrix factorization. The average affinity of a drug/target captures their typical binding behavior, which is valuable to predict the binding affinity with a specific target/drug. While *SimBoost* substantially outperforms matrix factorization, arguably because of its ability to discover non-linear relationships, the latent factors learnt by MF do provide important features for *SimBoost*.

## Conclusions

The majority of the existing cheminformatics methods for predicting drug–target interactions perform binary classification into binding and non-binding. In this paper we proposed the novel read-across method *SimBoost* for the problem of predicting continuous, as opposed to binary, drug–target binding affinities. As discussed above, continuous values allow the distinction between true negatives and missing values, and provide more information about the actual strength of protein-compound binding. To the best of our knowledge, *SimBoost* is the first non-linear method for continuous drug–target interaction prediction, and the first method that also computes a prediction interval as a measure of the confidence of the prediction.

In our experiments we compared *SimBoost* with *KronRLS*, the state-of-the-art method for this task, on the Davis, Metz, and KIBA datasets. To the best of our knowledge, this is the first study where the *KIBA* dataset was used for the evaluation of drug–target interaction predictions, and we believe that it should be used in future studies as a benchmark set, because of its heterogeneous and well-balanced nature.

In summary, our contributions are as follows:

- We proposed the first non-linear read-across method *SimBoost* for continuous drug–target binding affinity prediction. *SimBoost* takes informative features from the drug and target similarities and from a matrix factorization model, and trains a gradient boosting tree model.
- We proposed a version of *SimBoost*, called *SimBoostQuant*, which, using the same features, predicts binding affinities as well as prediction intervals as a measure of the confidence of the prediction.
- We performed extensive experiments on three datasets evaluating four performance metrics. The results demonstrate that *SimBoost* and *SimBoostQuant* consistently outperform state-of-the-art methods.

It should be noted that while *SimBoost* is a more accurate method, *SimBoostQuant* provides important information on the confidence of a prediction and, thus, explicitly address the Applicability Domain challenge. In our opinion, the choice between the two methods is essentially a trade-off between slightly more accurate versus somewhat more informative predictions.
